# Itaconic acid indicates cellular but not systemic immune system activation

**DOI:** 10.18632/oncotarget.25956

**Published:** 2018-08-14

**Authors:** Johannes Meiser, Lisa Kraemer, Christian Jaeger, Henning Madry, Andreas Link, Philipp M. Lepper, Karsten Hiller, Jochen G. Schneider

**Affiliations:** ^1^ Cancer Research UK Beatson Institute, Glasgow, UK; ^2^ University of Luxembourg, Luxembourg Centre for Systems Biomedicine, Luxembourg City, Luxembourg; ^3^ Saarland University Medical Centre, Centre of Experimental Orthopaedics, Homburg, Germany; ^4^ Saarland University Medical Centre, Department of Internal Medicine II, Homburg, Germany; ^5^ Saarland University Medical Centre, Department of Internal Medicine V, Homburg, Germany; ^6^ Braunschweig Integrated Centre of Systems Biology, Technische Universität Braunschweig, Braunschweig, Germany; ^7^ Department of Computational Biology of Infection Research, Helmholtz Centre for Infection Research, Braunschweig, Germany; ^8^ Centre Hospitalier Emile Mayrisch, Esch, Luxembourg

**Keywords:** itaconic acid, metabolism, sepsis, biomarker, inflammation, Immunology

## Abstract

Itaconic acid is produced by mammalian leukocytes upon pro-inflammatory activation. It appears to inhibit bacterial growth and to rewire the metabolism of the host cell by inhibiting succinate dehydrogenase. Yet, it is unknown whether itaconic acid acts only intracellularly, locally in a paracrine fashion, or whether it is even secreted from the inflammatory cells at meaningful levels in peripheral blood of patients with severe inflammation or sepsis.

The aim of this study was to determine the release rate of itaconic acid from pro-inflammatory activated macrophages *in vitro* and to test for the abundance of itaconic acid in bodyfluids of patients suffering from acute inflammation.

We demonstrate that excretion of itaconic acid happens at a low rate and that it cannot be detected in significant amounts in plasma or urine of septic patients or in liquid from bronchial lavage of patients with pulmonary inflammation.

We conclude that itaconic acid may serve as a pro-inflammatory marker in immune cells but that it does not qualify as a biomarker in the tested body fluids.

## INTRODUCTION

Recent reports have suggested itaconic acid (IA) to be a novel metabolic biomarker [1]. This suggestion is based on its intracellular identification in activated pro-inflammatory macrophages [2, 3] and the detection of IA in the plasma of pregnant women who developed diabetes mellitus after birth compared to controls not progressing to overt diabetes mellitus [1]. IA is a metabolic product derived from the tricarboxylic acid (TCA) cycle intermediate *cis*-aconitic acid, catalysed by *cis*-aconitate decarboxylase (IRG1/CAD). Two physiological functions of IA have been described in pro-inflammatory cells: (1) IA shows antibacterial properties by inhibiting bacterial isocitrate lyase, an enzyme of the glyoxylate shunt, giving microorganisms a survival advantage at carbohydrate limiting conditions [2, 4] and (2) IA inhibits succinate dehydrogenase (SDH) in the host cell to induce metabolic rewiring during pro-inflammatory activation [5, 6]. Hence, IA plays a dual complementary role by triggering metabolic adaptation of the host cell and by inhibiting growth of invading pathogens. It has been reported that IA is hardly detectable in quiescent macrophages but increases to millimolar concentrations upon pro-inflammatory activation within a few hours post-activation to a maximum level at ten hours post-activation [2, 6].

It has also been reported that IA is released from pro-inflammatory macrophages [6–8]. These reports suggest that IA might also be detectable in the blood of patients with bacterial sepsis and thus may possibly qualify as an early biomarker in a clinical setting. However, absolute quantifications of IA are so far lacking and the potential effects of extracellular IA are unclear to date [9].

To test the hypothesis if IA might be an early sepsis marker, we quantified IA in the extracellular space of macrophages *in vitro* and analysed different body fluids of septic patients.

## RESULTS

### IA release rates *in vitro*

**Figure 1:**
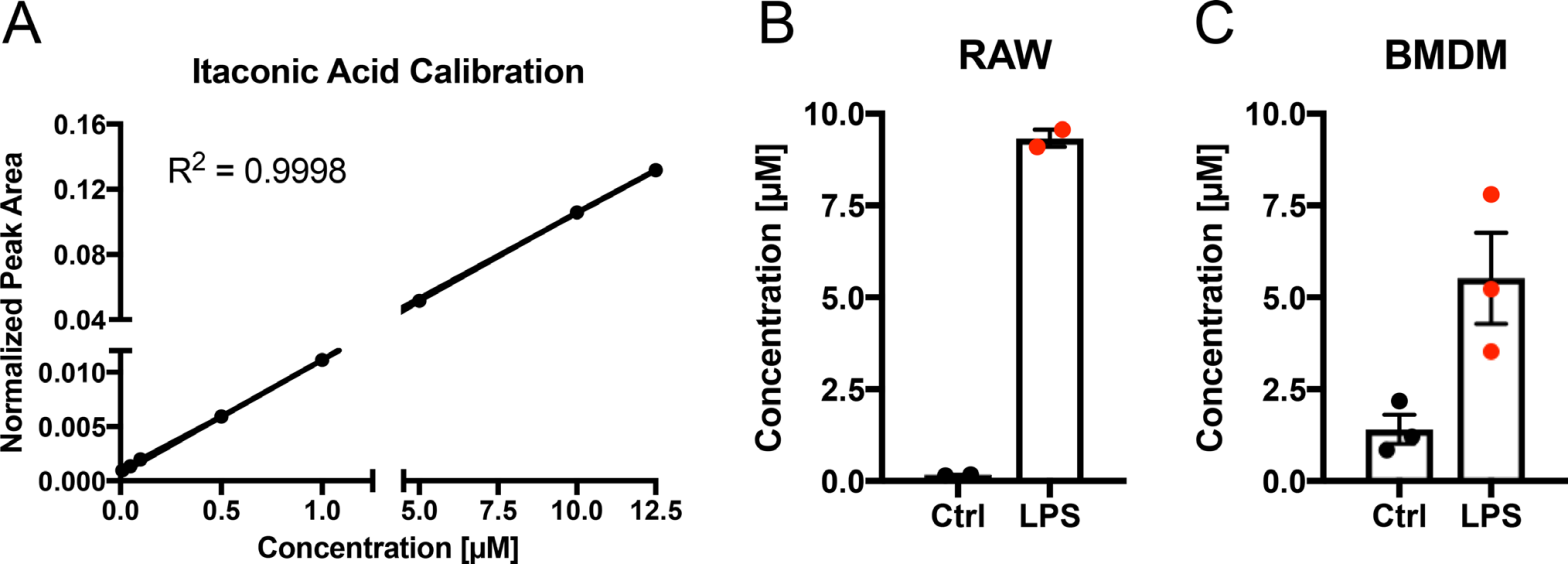
Quantification of itaconic acid concentration in cell culture medium upon LPS stimulation (**A**) External calibration curve (concentration range: 0.01–12.5 µM) for absolute quantification of IA in cell culture medium. (**B**–**C**) IA concentration (µM) in cell culture media of RAW264.7 (B) mouse macrophages and BMDMs (C) 6 h after stimulation (LPS) with 10 ng/ml LPS or of untreated cells (Ctrl). Each data point indicates one independent experiment with triplicate wells (2 experiments in (B) and three experiments in (C)). Data is represented as mean ± SEM.

To quantify IA released from lipopolysaccharide (LPS) activated macrophages *in vitro,* we used the mouse macrophage cell line RAW 264.7 and murine bone marrow derived macrophages (BMDM), activated the cells with 10 ng/ml LPS and analysed the medium for the presence of IA. We determined an average extracellular concentration of 9 µM for RAW 264.7 and 5 µM for BMDMs, respectively (Figure 1A–1C). Given the number of viable cells we estimated an average net release rate of 2.34 fmol/cell/h in the case of RAW264.7 cells and 0.53 fmol/cell/h in the case of BMDMs (Table 1). The determined differences in release rates are in line with the quantified intracellular IA concentrations (8 mM for RAW 264.7 cells [2] and 1.5 mM for BMDMs, respectively (Table 1)). Assuming passive diffusion as the main driving force for extracellular IA accumulation, higher intracellular concentrations result also in higher medium concentrations after LPS stimulation.

**Table 1:** Intracellular concentration of IA and release rates upon LPS activation

	Conc [mM]	Release [fmol/cell/h]
RAW	8 [2]	2.34
BMDM	1.51	0.53

The concentration in RAW cells is derived from [2]. Release rates were determined based on medium concentration (Figure 1), viable cells and time after LPS activation.

### Analysis of patient derived samples

The specific production of IA upon pro-inflammatory stimuli as well as the release of IA prompted us to test whether IA may potentially be measured in body fluids of septic patients or in patients with suspected progression of occult systemic bacterial infection to overt sepsis. In this case IA could represent a novel biomarker to improve to the existing clinical and laboratory portfolio for inflammation assessment.

Therefore, we analysed blood and urine samples of different sepsis patients (Table 2), bronchoalveolar lavage fluids (BALF) of patients with different lung diseases including bacterial infection or exacerbation of chronic pulmonary disease (Table 3).

**Table 2:** Sepsis patient overview

Gender	Age	Type of Infection	Leu	CRP	PCT	Type and duration of Therapy
M	60	Unclear	8.7	117	8.7	Fortum and Tygacil 5th day
M	75	Retention pneumonia	14.3	101	1.6	Tazobac, Meronem 2nd day
M	57	Pancreatitis and Pneumonia	6.8	390	16.6	Tazobac, Tavanic 2nd day
M	71	Pneumonia (repeated sepsis)	9.4	133	1.1	Vancomycin, Meronem 1st day
M	64	Aspiration pneumonia,	13.9	332	2.5	Vancomycin, Meronem 1st day
M	57	Endocarditis, pneumonia	18.9	377	2.1	Zyvoxid, Eremfat 1st day, Clont 3rd day
M	57	Pneumonia	9	111	1.1	Tazobac, Tavanic 1st day
M	73	Retention pneumonia, STEMI	7.4	130	2.4	Tazobac, Tavanic 2nd day
M	59	Retention pneumonia, cardiogenic shock	11	234	3.5	Vancomycin, Meronem 1st day
M	57	Pneumonia ARDS	18.7	81	2.4	Maxipime, Vancomycin 4th day
M	86	Retention pneumonia	17.7	173	1.4	Vancomycin, Meronem 2nd day
M	66	Urosepsis	22	402	122	Tazobac, Tavanic 2nd day
M	70	Retention pneumonia	19.5	188	1.3	Vancomycin, Meronem 2nd day
F	85	Pneumonia (aspiration pneumonia)	7.1	208	4.8	Tazobac, Tavanic 4th day
M	77	Pneumonia, infected hematoma	10.8	315	2.2	Vancomycin, Meronem 2nd day
M	68	Pneumonia	14.2	211	9.1	Vancomycin, Meronem, Clont 2nd day
M	66	Aspiration pneumonia	10.5	149	5.6	Tazobac, Tavanic 2nd day
M	55	Retention pneumonia during cardiogenic shock	19.6	270	4.7	Tazobac, Cubicin 2n day
F	N/A	Urosepsis	20.9	201	262	Vancomycin, Meronem 1st day

Healthy individuals served as controls. PCT: pro-calcitonin; CRP: c-reactive protein.

**Table 3:** Patient overview bronchial lavage samples

gender	Age	leucocytes (G/L)	CRP (mg/L)	pathology
f	56	11,80	42,30	pulmonary abscess
m	67	6,60	26,30	interstitial nephritis
m	44	6,40	9,80	pneumonia
m	63	8,30	3,30	COPD
f	44	14,20	1,70	bronchogenic cyst
m	54	24,50	5,60	hemoptysis
f	52	2,10	17,80	adenocarcinoma
f	27	8,90	n.a.	sarcoidosis
f	62	6,60	1,20	adenocarcinoma
m	77	6,40	4,50	adenocarcinoma
f	62	10,00	3,10	small cell lung cancer
m	69	1,04	32,90	mesothelioma
f	69	7,20	8,90	small cell lung cancer
f	69	n.a.	n.a.	chronic cough
m	74	7,90	24,00	pneumonia
f	77	7,80	23,10	adenocarcinoma

**Figure 2:**
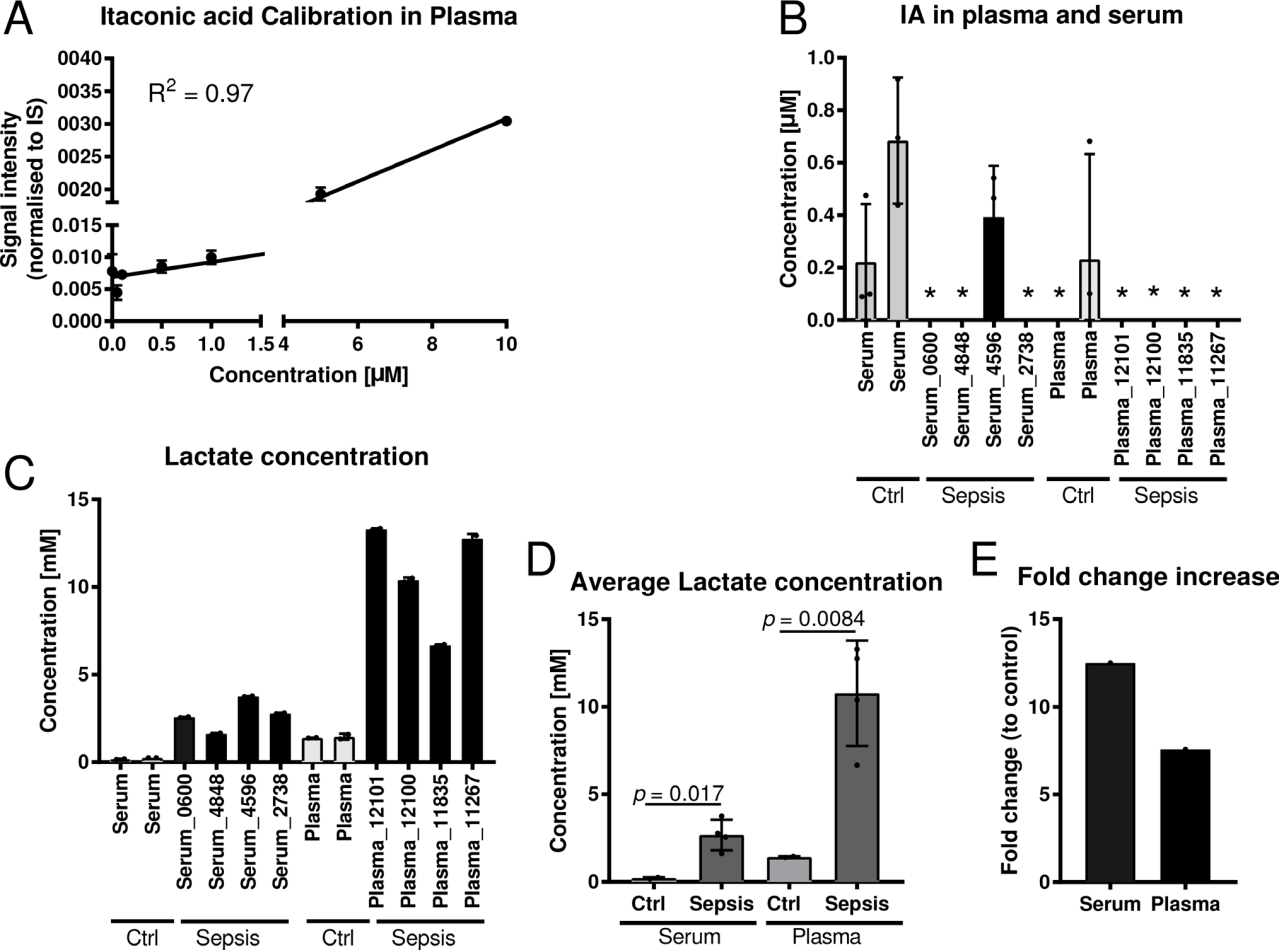
Quantification of itaconic acid and lactic acid in serum and plasma samples of sepsis patients (**A**) External calibration curve (concentration range: 0.01–10 µM) for absolute quantification of IA in plasma and serum. (**B**) Quantification of IA in four plasma and four serum samples of sepsis patients with respective healthy control samples. For background subtraction the determined average IA concentration of control samples was subtracted of the determined concentrations of the sepsis samples. Each sample was analysed in three technical replicates. ^*^indicates samples with IA concentrations below the detection limit. (**C**–**D**) Quantification of lactate in the same samples as in (B) and (**E**) respective fold change increase of lactate in sepsis samples compared to respective controls. Data (A–D) is represented as mean ± SD.

For the analysis, we first determined the limit of quantification (LOQ) for IA in plasma with our analytical pipeline, using gas chromatography coupled to mass spectrometry (GC-MS). With 1 µM (Figure 2A) the LOQ in plasma was higher compared to medium (Figure 1A) but reasonable, since the high protein content of plasma may bind a significant fraction of available metabolites, resulting in low recovery of IA. Then, we applied our analysis pipeline to screen the collected body fluids for IA. We did not detect any signal for IA in any of the body fluids, except for one single patient where we detected a low signal below the limit of quantification in the urine. However, we did not detect a signal in the respective plasma sample. Interestingly, this patient was suffering from a genitourinary tract infection and urosepsis.

To cross validate our results we employed a set of commercial sepsis samples comprising four plasma and four serum samples (Table 4). Also in these samples, all values were close to, or below the detection limit (Figure 2B). As a positive control we also measured lactate concentrations, a metabolite that is known to be increased under septic conditions. As expected, both serum and plasma samples had significantly increaseed lactate concentrations compared to respective controls (Figure 2C–2E).

**Table 4:** Overview commercially obtained blood samples of septic patients

Product ID	Matrix	Age	Gender	Ethnicity	Type of Infection
KH17_11267	K2 EDTA Plasma	86	M	White	Beta-hemolytic streptococci group G
KH17_11835	K2 EDTA Plasma	84	F	White	E.coli
KH17_12100	K2 EDTA Plasma	65	F	White	H.influenza
KH17_12101	K2 EDTA Plasma	69	F	White	Klebsiella pneumoniae
KH17_2738	Serum	82	F	White	E.coli
KH17_4848	Serum	45	M	White	Klebsiella pneumoniae
KHBR_0600	Serum	42	M	Black	Staph haemolyticus
KH17_4596	Serum	53	M	Black	Staph epidermidis

**Figure 3:**
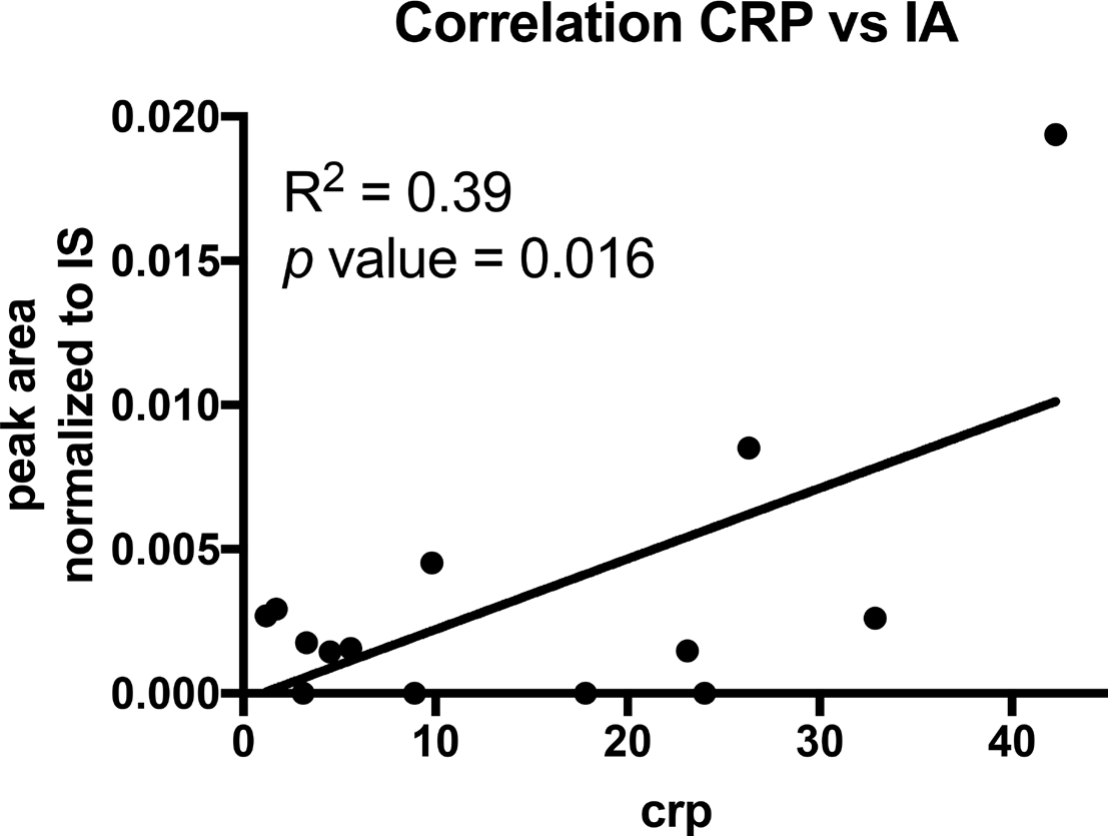
Correlation analysis of CRP and IA signals in cell pellets of BALF’s Each dot indicates one patient (*n* = 14). *p* value was calculated using linear regression.

Intrigued by the one urine sample where we observed at least a low IA signal, we further investigated if patient derived cells contain IA and thus, locally, leukocytes might increase IA concentrations by low excretion rates. To that end, we analysed the cell pellets obtained from the bronchial lavages. In patients with severe lung infections, leukocytes migrate into the alveoli and are aspirated during the lavage. These cells were pelleted during sample processing and snap frozen. Intracellular metabolites of these pellets were extracted and analysed with GC-MS. In most of these samples we could detect low abundant signals for IA and we observed a significant correlation (*R*^2^ = 0.39; *p*-value = 0.016) with the C-reactive protein (CRP) values (Figure 3, Table 3), indicating that systemic inflammation correlates with the detected IA signals. Hence, it might be possible that locally, IA concentrations in the microenvironment are increased as a result of low excretion rates.

## DISCUSSION

In this study we profiled IA release rates from macrophages *in vitro* and we analysed different body fluids of patients suffering from acute sepsis to test if IA might accumulate to sufficient concentrations in the circulation. The latter case would mean that IA could potentially serve as a novel biomarker in clinical settings to detect systemic bacteremia [10]. A novel metabolic biomarker detectable in the plasma could be an asset to the existing clinical and laboratory portfolio for sepsis assessment, since a precise diagnosis for acute sepsis, especially at an early stage, is still challenging [11]. Using marker metabolites in blood samples as biomarkers is of high interest due to the ease of accessibility and availability in clinical settings. A well-known example is the oncometabolite 2-hydroxyglutarate (2HG), which can be used as a biomarker in isocitrate dehydrogenase (IDH) mutated acute myeloid leukemia (AML) and intrahepatic cholangiocarcinoma (ICC) patients [12, 13]. Due to its rapid production upon extracellular pro-inflammatory stimuli, IA could theoretically qualify as an early biomarker, helping to identify minimal systemic inflammatory responses similar or even superior to pro-calcitonin [14]. A similar hypothesis has recently been raised in the context of rheumatoid arthritis [15]. However, we were not able to detect elevated IA levels in the analysed body fluids from septic patients. Therefore, IA does not seem to be a suitable biomarker of systemic inflammation employing standard routine laboratory methodology.

Our result is in contrast to the previous report by de Seymour *et al.* [1], who reported IA as a biomarker in peripheral blood of pregnant women who progressed to overt diabetes mellitus *post partum*. The reason for this discrepancy in detecting IA in peripheral blood remains unclear and might be at best caused by different technology being applied for detection. However, our results clearly indicate that in a clinical setting, IA is not detectable with a standard (but state-of-the-art) analytical infrastructure even in septic conditions. This observation is corroborated by the fact that there is, to our knowledge, no other report of increased IA concentrations, neither in sepsis nor other patient cohorts.

The low concentrations of IA in the circulation are supported by the very low excretion rates that we determined in our *in vitro* experiments. Despite high intracellular IA concentrations in the mM range we only observed very low release rates. As a comparison, the extracellular lactate concentration in similar experiments with RAW 264.7 cells has been reported to be 20 mM, resulting in a net release rate of 170 fmol/cell/h [16], indicating that the release of IA into the extracellular space happens at a low rate.

The IA concentration in the tissue culture media might also be explained by accumulative release from apoptotic cells or by lysosomal trafficking and fusion with the plasma membrane. However, the latter mechanism alone is not sufficient to reach the measured concentration in our *in vitro* experiments, suggesting that the detected IA might be a net result of both, diffusion and release from vesicles and apoptotic cells. As an alternative to release, IA might be further metabolised within the cell although a mammalian metabolic degradation pathway for IA has so far only been described for the liver [17, 18]. To provide detailed explanation on these possibilities, further work is required in the future.

The notion that IA is mainly intracellularly abundant and not actively excreted is supported by our observation that we could not detect IA in the liquids of BALF’s but in intracellular extracts of BALF derived cell pellets.

When analysing the intracellular extracts, we even observed a correlation between the measured IA signal and the determined CRP values. These data suggest that the higher IA signals originate from the intracellular space of the leukocytes associated with the increased CRP. We can speculate that this specific observation also applies to the recorded signal in the urine of the urosepsis patient where the IA signal likely derived from apoptotic leukocytes in urine.

Based on the low excretion rates, a paracrine effect in the microenvironment is still debatable. But due to its chemical properties as a charged molecule IA can hardly cross the plasma membrane, which is why in *in vitro* experiments extracellular IA is applied in the mM range to cross the plasma membrane and/or membrane permeable dimethylIA is used [5, 6]. In terms of potential antibacterial properties in the extracellular space, IA concentrations are most probably orders of magnitude too low to inhibit bacterial growth [2]. Required concentrations in the mM range are most likely only possible to appear within the cells. We still cannot exclude that IA may have distinct effects involving the activation of so far unknown signalling pathways or facilitating receptor activation. An example for such a metabolite derived activation, with similar chemical structure, is given by succinate activating GPR91 to promote stem cell migration [19]. However, at this point testing such hypotheses are beyond the scope of the present manuscript. Our data support primarily the notion that IA acts as a cell intrinsic factor to inhibit SDH in the host cell and potentially, to support bacterial killing in lysosomes by inhibiting the glyoxylate shunt.

To reach a translational pre-clinical stage it will be important to evaluate if and how IRG1/CAD and IA could be exploited to treat inflammatory diseases in humans rather than serving as biomarkers.

In summary, our results indicate that IA may rather acts as a local antimicrobial metabolite. It does not represent a suitable biomarker to be used in a clinical setting.

## MATERIALS AND METHODS

### Cell culture

RAW 264.7 mouse leukaemic cells (ATCC^®^TIB-71™) were cultured as described in detail in [16]. Briefly, cells were cultured in DMEM D5796 supplemented with 10% FBS. Macrophages (BMDM) were differentiated from bone marrow of wild type mice: Bone marrow was flushed and isolated using ice-cold PBS. Cells were then cultured in DMEM D5796 supplemented with 10% FBS, 10% M-CSF and 0.5% Pen/Strep. At day 5, adherent macrophages were collected by scraping, counted and seeded for experiment. LPS (from *E.coli*, Sigma L6529) was prepared as a working stock of 1 μg/mL in DMEM and spiked 1:100 into the wells of the culture dish for a final concentration of 10 ng/mL. For cell counting and assessment of cell viability a Vi-Cell™ XR (Beckman Coulter) automated cell counter was used according to the manufacturer’s instructions.

### Metabolite extraction from medium, derivatisation, and GC-MS measurement

The method was adopted from [20] with the following changes: Extracellular metabolites from culture medium samples were extracted in triplicate using a methanol/water mixture (5:1, v/v). The water fraction contained the internal standard (IS) Pentanedioic acid-D6 (*c* = 10 µg/mL; C/D/N Isotopes Inc.). 40 µL of medium were added to 240 µL of the ice-cold methanol/water+IS mixture (5:1, v/v). After adding 100 µL ice-cold chloroform, the mixture was vortexed for 10 min at 4° C and 2,000 rpm (Eppendorf Thermomixer C). For phase separation, 100 µl of chloroform and 100 µl of water were added and vortexed for 1 min at 4° C and 2,000 rpm. Then, the mixture was centrifuged at 21,000 × g for 5 min at 4° C. 250 µL of the polar (upper) phase were transferred to GC glass vial with micro insert (5–250 µL) and evaporated to dry under vacuum at −4° C.

For absolute metabolite quantification, a dilution series of IA was included in the extraction procedure and measured in triplicate. The stock solution (*c* = 20 mM) was freshly prepared by dissolving IA in water. It was used for the preparation of an external calibration curve in DMEM D5796 with 10% FBS medium with the following concentration levels: 0.01; 0.05; 0.1; 0.5; 1; 5; 10 and 12.5 µM.

Metabolite derivatisation was performed by using a multipurpose sampler (Gerstel). Dried medium extracts were dissolved in 15 µL pyridine, containing 20 mg/ml methoxyamine hydrochloride (Sigma-Aldrich), at 55° C for 90 min under shaking. After adding 15 µL MTBSTFA + 1% TBDMSCI (Restek), samples were incubated at 55° C for 60 min under continuous shaking.

GC-MS analysis was performed by using an Agilent 7890A GC coupled to an Agilent 5975C inert XL Mass Selective Detector (Agilent Technologies). A sample volume of 1 µL was injected into a Split/Splitless inlet, operating in split mode (10:1) at 270° C. The gas chromatograph was equipped with a 30 m (I.D. 0.25 mm, film 0.25 µm) DB-35MS capillary column (Agilent J&W GC Column). Helium was used as the carrier gas with a constant flow rate of 1.4 mL/min. The GC oven temperature was held at 100° C for 1 min and increased to 200° C at 8° C/min. Then, the temperature was increased to 325° C at 50° C/min and held for 6 min. The total run time was 22 min. The transfer line temperature was set to 280° C. The MSD was operated under electron ionization at 70 eV. The MS source was held at 230° C and the quadrupole at 150° C. For precise quantification, GC-MS measurements were performed in selected ion monitoring (SIM) mode using the following masses: *m/z* 301.2; 343.2; 358.2 (dwell times: 60 ms) for itaconic acid and *m/z* 235.2; 309.2; 351.3 (dwell times: 75 ms) for Pentanedioic acid-D6.

### Metabolite extraction from plasma, serum and urine, derivatisation, and GC-MS measurement

200 µL urine were centrifuged at 400 × g for 5 min at 4° C. 100 µL supernatant were mixed with 75 µL urease (5 mg/mL in HEPES, *c* = 25 mM, pH: 7,0). The mixture was kept for 5 min at 37° C. 15 µL were mixed with 135 µL of methanol/water mixture (8:1, v/v). The mixture was vortexed for 5 min at 4° C and 1,400 rpm (Eppendorf Thermomixer). Then, the mixture was centrifuged at 21,000 × g for 5 min at 4° C. 30 µL were transferred to a GC glass vial with micro insert (5–250 µL) and evaporated to dry under vacuum at –4° C.

For plasma and serum extraction, 15 µL of plasma or serum were mixed with 135 µL of methanol/water mixture (8:1, v/v) including internal standard Pentanedioic acid-D6 (*c* = 2 µg/mL). Further processing was identical to the urine samples. For commercial sepsis samples, 60 µL were transferred to a GC glass vial with micro insert and evaporated to dry under vacuum at –4° C.

Metabolite derivatisation was performed by using a multipurpose sampler (Gerstel). Dried medium extracts were dissolved in 15 µL pyridine, containing 20 mg/mL methoxyamine hydrochloride (Sigma-Aldrich), at 40° C for 60 min under shaking. After adding 15 µL MSTFA (Macherey-Nagel), samples were incubated at 40° C for 30 min under continuous shaking.

GC-MS analysis was performed by using an Agilent 7890A GC coupled to an Agilent 5975C inert XL Mass Selective Detector (Agilent Technologies). A sample volume of 1 µL was injected into a Split/Splitless inlet, operating in splitless mode (plasma split mode 10:1) at 270° C. The gas chromatograph was equipped with a 30 m (I.D. 250 µm, film 0.25 µm) DB-35MS capillary column (Agilent J&W GC Column). Helium was used as the carrier gas with a constant flow rate of 1.2 mL/min. The GC oven temperature was held at 80° C for 1 min and increased to 320° C at 15° C/min and held for 8 min. The total run time was 25 min. Plasma: 90° C for 1 min, 9° C/min to 270° C, 25° C/min to 320° C. Held for 7 min. The total run time was 30 min. The transfer line temperature was set to 280° C. The MSD was operated under electron ionization at 70 eV. The MS source was held at 230° C and the quadrupole at 150° C. For precise quantification, GC-MS measurements were performed in SIM mode using the following masses: *m/z* 215.1; 230.1; 259.1 (dwell times: 50 ms) for Itaconic acid and m/z 206.1; 239.1; 267.1 (dwell times: 50 ms) for Pentanedioic acid-D6.

### Metabolite extraction from bronchioalveolar lavage fluid (BALF), derivatisation, and GC-MS measurement

For BALF extraction, 15 µL of BALF were mixed with 135 µL of methanol/water mixture (8:1, v/v) including internal standard Pentanedioic acid-D6 (*c* = 2 µg/mL). The mixture was vortexed for 5 min at 4° C and 1,400 rpm (Eppendorf Thermomixer). Then, the mixture was centrifuged at 21,000 × g for 5 min at 4° C. 60 µL were transferred to a GC glass vial with micro insert and evaporated to dry under vacuum at −4° C.

Metabolite derivatisation was performed by using a multipurpose sampler (Gerstel). Dried medium extracts were dissolved in 15 µL pyridine, containing 20 mg/mL methoxyamine hydrochloride (Sigma-Aldrich), at 55° C for 90 min under shaking. After adding 15 µL MTBSTFA + 1% TBDMSC (Restek), samples were incubated at 55° C for 60 min under continuous shaking.

GC-MS analysis was performed by using an Agilent 7890A GC coupled to an Agilent 5975C inert XL Mass Selective Detector (Agilent Technologies). A sample volume of 1 µL was injected into a Split/Splitless inlet, operating in splitless mode at 270° C. The gas chromatograph was equipped with a 30 m (I.D. 0.25 mm, film 0.25 µm) DB-35MS capillary column (Agilent J&W GC Column). Helium was used as the carrier gas with a constant flow rate of 1.2 mL/min. The GC oven temperature was held at 100° C for 2 min and increased to 300° C at 10° C/min. Then, the temperature was held for 4 min. The total run time was 26 min. The transfer line temperature was set to 280° C. The MSD was operated under electron ionization at 70 eV. The MS source was held at 230° C and the quadrupole at 150° C. For precise quantification, GC-MS measurements were performed in selected ion monitoring (SIM) mode using the following masses: *m/z* 301.2; 343.2; 358.2 (dwell times: 60 ms) for Itaconic acid and *m/z* 235.2; 309.2; 351.3 (dwell times: 75 ms) for Pentanedioic acid-D6.

### Metabolite extraction and intracellular itaconic acid quantification from BMDMs

Prior extraction, LPS-stimulated and non-stimulated BMDMs were washed with 0.9% NaCl. For intracellular metabolite extraction, 200 µL of ice-cold methanol and 200 µL of water containing 1 µg/mL Pentanedioic acid-D6 as internal standard were added and cells were scraped and transferred into a 1.5 mL reaction tube containing 200 µl of chloroform. For intracellular Itaconic acid quantification by standard addition, aliquots of the water fraction (containing internal standard) were separately spiked with different concentrations of Itaconic acid (25; 50 and 100 µM) to extrapolate intracellular Itaconic acid levels. The mixtures were vortexed for 20 min at 4° C and 1,400 rpm and centrifuged for 10 min at 4° C and 17,000 × g. 200 µL of the upper polar phase were transferred to a GC glass vial with micro insert and evaporated to dry under vacuum at –4° C. Derivatization and GC-MS measurement was performed as reported in section “Metabolite extraction from bronchioalveolar lavage fluid (BALF), derivatisation, and GC-MS measurement”.

### Metabolite extraction from BALF cell pellets

For metabolite extraction from cells present in BALF, 200 µl ice-cold methanol, 200 µL water containing 1 µg/mL Pentanedioic acid-D6 as internal standard and 200 µL chloroform was added to the cell pellet and vortexed for 20 min at 4° C and 1,400 rpm. Then, the mixture was centrifuged for 10 min at 4° C and 17,000 × g and 250 µL of the upper polar phase were transferred to a GC glass vial with micro insert. Extracts were dried under vacuum at −4° C. Derivatization and GC-MS measurement was performed as described in section “Metabolite extraction from bronchioalveolar lavage fluid (BALF), derivatisation, and GC-MS measurement”.

### Data normalisation and data processing

All GC-MS chromatograms were processed using MetaboliteDetector, v3.020151231Ra [21]. The software package supports automatic deconvolution of all mass spectra. Compounds were annotated by retention time and mass spectrum. The internal standard was added at the same concentration to every medium sample to correct for uncontrolled sample losses and analyte degradation during metabolite extraction. The data set was normalised by using the response ratio of the integrated peak area: analyte and the integrated peak area:_internal standard. Absolute concentrations were determined using the response ratios of the calibration curve of Itaconic acid as a function to quantify concentrations.

### Patient study

The cohort study was approved by the ethics committee of the state of Saarland, Germany (230/11) as well as the state research ethics committee of Luxembourg (201203/07). Patients or legal guardian were informed and asked for consent before sampling took place. Plasma and urine samples were collected from patients that were hospitalised for sepsis. Diagnosis was based on chart information (fever, c-reactive protein elevation CRP, evidence of bacteria in bloodstream, antibiotic treatment). Sampling procedures were incorporated into routine blood, urine or bronchial lavage examinations to avoid any extra stress to the subjects. Clinical information was taken from the chart. Blood and urine samples were immediately processed as described above.

Commercial sepsis samples were purchased from Discovery Life Sciences (for sample details see Table 4).

## Abbreviations

CRP: C reactive protein
GC-MS: Gas Chromatography Mass Spectrometry
IA: Itaconic acid
Leu: Leukocytes
LPS: lipopolysaccharide
PCT: Procalcitonin
SDH: Succinate dehydrogenase
SIM: Selected Ion Monitoring
